# Fungal Cell Wall: Emerging Antifungals and Drug Resistance

**DOI:** 10.3389/fmicb.2019.02573

**Published:** 2019-11-21

**Authors:** Soraia L. Lima, Arnaldo L. Colombo, João N. de Almeida Junior

**Affiliations:** ^1^Laboratório Especial de Micologia, Disciplina de Infectologia, Universidade Federal de São Paulo, São Paulo, Brazil; ^2^Central Laboratory Division, Hospital das Clínicas da Faculdade de Medicina da Universidade de São Paulo, São Paulo, Brazil

**Keywords:** fungal cell wall, antifungals, therapy, resistance, 1,3-β-D-Glucan Synthase Inhibitors, ibrexafungerp, manogepix

## Abstract

The cell wall is an essential component in fungal homeostasis. The lack of a covering wall in human cells makes this component an attractive target for antifungal development. The host environment and antifungal stress can lead to cell wall modifications related to drug resistance. Antifungals targeting the cell wall including the new β-D-glucan synthase inhibitor ibrexafungerp and glycosyl-phosphatidyl Inositol (GPI) anchor pathway inhibitor fosmanogepix are promising weapons against antifungal resistance. The fosmanogepix shows strong *in vitro* activity against the multidrug-resistant species *Candida auris*, *Fusarium solani*, and *Lomentospora prolificans.* The alternative carbon sources in the infection site change the cell wall β-D-glucan and chitin composition, leading to echinocandin and amphotericin resistance. *Candida* populations that survive echinocandin exposure develop tolerance and show high chitin content in the cell wall, while fungal species such as *Aspergillus flavus* with a higher β-D-glucan content may show amphotericin resistance. Therefore understanding fungal cell dynamics has become important not only for host-fungal interactions, but also treatment of fungal infections. This review summarizes recent findings regarding antifungal therapy and development of resistance related to the fungal cell wall of the most relevant human pathogenic species.

## Introduction

The cell wall is an essential component in homeostasis of fungal cells (Latgé, 2007; Gow et al., 2017). It also has a dual interaction process with the surrounding environment, which either negatively or positively impacts fungal cell survival. Cell wall antigens induce immune recognition by the infected host and facilitate phagocytosis (Roy and Klein, 2012). Some antigens, named pathogen-associated molecular patterns (PAMPs), are recognized by a wide range of pattern-recognition receptors (PRRs) on host cell surfaces (Roy and Klein, 2012). Conversely, environmental stresses lead to cell wall modifications that impede immune recognition (Gow et al., 2017).

Representing approximately 40% of the total fungal cell volume, the fungal cell wall forms a tensile and robust core scaffold to which a variety of proteins and superficial components with fibrous and gel-like carbohydrates form polymers, making a strong but flexible structure (Munro, 2013; Gow et al., 2017). Most cell walls have two layers: (1) the inner layer comprising a relatively conserved structural skeleton and (2) the outer layer which is more heterogeneous and has species-specific peculiarities (Gow et al., 2017). The inner cell wall represents the loadbearing, structural component of the wall that resists the substantial internal hydrostatic pressure exerted on the wall by the cytoplasm and membrane (Latgé, 2007). This layer includes chitin and glucan, in which 50–60% of the dry weight of the cell wall is made up of β-(1-3)-glucan. The outer-layer structure consists of heavily mannosylated glycoproteins with modified N- and O- linked oligosaccharides. The structure of these oligosaccharide side chains differs among fungal species (Shibata et al., 1995; Hobson et al., 2004).

Since human cells do not have a covering wall, antifungals that target the production of cell wall components are more selective and less toxic when compared to azole derivatives and amphotericin B (Patil and Majumdar, 2017). Echinocandins were the first systemic antifungals that targeted the cell wall by disrupting the production of glucans (Patil and Majumdar, 2017). For invasive candidiasis, echinocandins were a great development that lowered the mortality associated with these infections, with low toxicity and few interactions with other medication (Mora-Duarte et al., 2002; Pappas et al., 2016). However, intrinsic and acquired resistance to echinocandins limits its usefulness, leading to research into other targets in the fungal cell wall for antifungal therapy (Hasim and Coleman, 2019).

Cell wall dynamics may play an important role for the development of antifungal resistance and interesting concepts regarding this subject are emerging. Structural and cell wall composition modifications have been investigated in *Candida* and *Aspergillus* isolates presenting antifungal resistance (Seo et al., 1999; Mesa-Arango et al., 2016). In echinocandin-tolerant *Candida* isolates, β-1,3- and β-1,6-glucans crosslinks modifications and higher chitin content have been described (Perlin, 2015), while higher β-D-glucan composition has been found in amphotericin-B-resistant *Aspergillus flavus* isolates (Seo et al., 1999).

In this manuscript, we review the fungal cell wall as a target for antifungal therapy and, in conjunction, visit cell wall modifications that may be related to antimicrobial resistance.

## Fungal Cell Wall Targeting Antifungals

**Figure 1:**
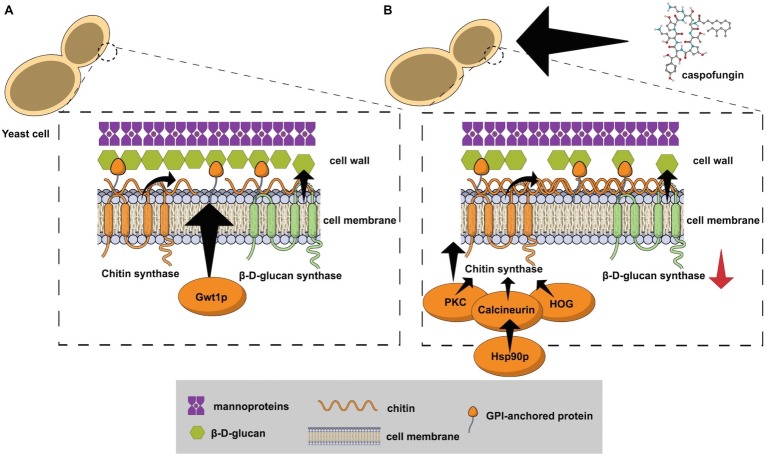
**(A)** The fungal cell wall and the targets that have been explored for antifungal development: β-D-glucan synthase, chitin synthase, and the enzyme Gwt1 from the GPI anchor pathway; **(B)** Echinocandin exposure causes cell wall stress by inhibition of the β-D-glucan synthase. The protein kinase C (PKC), high osmolarity glycerol response (HOG), and Ca^+2^-calcineurin pathways have been implicated in the response to cell wall damage and chitin synthase hyper stimulation. Calcineurin is a client protein for the Hsp90 chaperone and genetic compromise of the gene *HSP90* reduces the tolerance mechanism.

Antifungals targeting the cell wall have been developed in the last years (Walker et al., 2011; Chaudhary et al., 2013; Mutz and Roemer, 2016; Hasim and Coleman, 2019). Most of these drugs act by inhibiting β-D-glucan synthase, but chitin synthase and glycosylphosphatidylinositol (GPI) anchor pathway inhibitors are also under development (Figure 1A).

### 1,3-β-D-Glucan Synthase Inhibitors

#### Echinocandins

Echinocandins were first described in the 1970’s as antibiotic polypeptides obtained from *Aspergillus nidulans* (Nyfeler and Keller-Schierlein, 1974). These molecules are basically hexapeptide antibiotics with N-linked acyl fatty acid chains that intercalates with the phospholipid layer of the cell membrane (Denning, 2003). This antifungal class inhibits the β-D-glucan synthase, which leads to a decrease of the β-D-glucans in the cell wall after noncompetitively binding to the Fksp subunit of the enzyme (Hector, 1993; Denning, 2003; Aguilar-Zapata et al., 2015; Perlin, 2015; Patil and Majumdar, 2017).

The fungal cell wall β-D-glucan synthase complex has two main subunits: Fks1p and Rho1p (Mazur and Baginsky, 1996; Aguilar-Zapata et al., 2015). Fks1p is the catalytic subunit responsible for the production of glycosidic bonds (Schimoler-O’Rourke et al., 2003), while Rho1p is a Ras-like GTP-binding protein that regulates the β-D-glucan synthase activity (Qadota et al., 1996).

Inhibition of β-D-glucan synthase results in the cell death of the *Candida* species, while echinocandins modify the hyphae morphogenesis and exert a fungistatic effect against *Aspergillus* species (Bowman et al., 2002). Conversely, species belonging to the order Mucorales and the basidiomycetes are intrinsically resistant to this antifungal class (Espinel-Ingroff, 2003; Aguilar-Zapata et al., 2015).

Currently, there are three echinocandins approved by the FDA for the treatment of invasive fungal infections: caspofungin, anidulafungin, and micafungin (Johnson and Perfect, 2003; Rüping et al., 2008; Pappas et al., 2016). Compared to other antifungal classes, the echinocandins show lower kidney or liver toxicity, fewer drug–drug interactions, and have predominant liver elimination, not requiring dose adjustments during renal failure or dialysis (Aguilar-Zapata et al., 2015). However, echinocandins have pharmacokinetic limitations, such as poor bioavailability upon oral administration, high protein binding, and low central nervous system (CNS) penetration (Wiederhold and Lewis, 2003). New glucan synthase inhibitors with better pharmacokinetics profiles, including oral formulations with high bioavailability, are under investigation (Davis et al., 2019).

Rezafungin (CD101, formerly SP3025, Cidara Therapeutics, San Diego, CA, USA), a next-generation echinocandin, is currently in Phase 3 of clinical trials for the treatment of candidemia and invasive candidiasis^1^. This antifungal is a structural analog of anidulafungin, but with a choline moiety replacing the hemiaminal group at the C5 ornithine position, resulting in a stable compound with prolonged half-life (Sandison et al., 2017). It is highly soluble in aqueous systems and has a half-life of over 130 h in humans, compared to 24, 9–11, 10–17 h half-lives of anidulafungin, caspofungin, and micafungin, respectively (Kofla and Ruhnke, 2011; Sandison et al., 2017). The long half-life of rezafungin allows an advantageous weekly dosing regimen (Sandison et al., 2017; Sofjan et al., 2018).

Rezafungin has potent *in vitro* activity against common *Candida* and *Aspergillus* species (Wiederhold et al., 2018; Arendrup et al., 2018a,b). Furthermore, this antifungal has strong *in vitro* antifungal activity against the potential multidrug-resistant species *C. auris* (Berkow and Lockhart, 2018). Moreover, the *in vivo* efficacy of rezafungin in neutropenic murine disseminated candidiasis models was demonstrated against *C. albicans, C. glabrata, C. parapsilosis* (Lepak et al., 2018), and *C. auris* (Hager et al., 2018a).

#### Triterpenoids

The triterpenoid class is represented by ibrexafungerp (SCY-078, formerly MK-3118), a new semisynthetic derivate of hemiacetal triterpene glycoside enfumafungin (Synexis Inc., Jersey City, NJ, USA) (Pfaller et al., 2017; Wring et al., 2017; Davis et al., 2019). It is a β-D-glucan synthase inhibitor with similar but not identical biding sites to echinocandins in the catalytic regions Fks1p and Fks2p of the enzyme (Walker et al., 2011; Jiménez-Ortigosa et al., 2017). It has high protein binding and good tissue penetration, although like echinocandins, it has poor CNS penetration (Davis et al., 2019). The pharmacokinetic gain of this new antifungal is its good oral bioavailability (Walker et al., 2011).

Ibrexafungerp has demonstrated good *in vitro* activity against relevant fungal pathogens such as *Candida* spp., including multidrug-resistant *C. glabrata* (Pfaller et al., 2013, 2017; Jiménez-Ortigosa et al., 2017), biofilm producer strains (Marcos-Zambrano et al., 2017b), and *C. auris* (Larkin et al., 2017). Notably, echinocandin-resistant *Candida* strains harboring hot spot mutations at the Fksp may retain susceptibility to ibrexafungerp (Pfaller et al., 2017). A more in-depth study analyzing *C. glabrata* strains with echinocandin resistance and ibrexafungerp susceptibility showed that ibrexafungerp has only partial overlapping at the echinocandins Fksp biding sites in the β-D-glucan synthase enzyme (Jiménez-Ortigosa et al., 2017). Against *Aspergillus* clinically relevant species, ibrexafungerp has also demonstrated potent *in vitro* activity (Davis et al., 2019). Moreover, the combination of ibrexafungerp with either voriconazole or amphotericin B has demonstrated synergy against wild-type *A. fumigatus* strains (Ghannoum et al., 2018). Noteworthy, ibrexafungerp showed some antifungal activity against the multidrug-resistant mold *Lomentospora prolificans* (Lamoth and Alexander, 2015), and it is highly active against *Paecilomyces variotii* (Lamoth and Alexander, 2015). However, ibrexafungerp has little activity against Mucorales spp., *Fusarium* spp., and *Purpureocillium lilacinum* (Lamoth and Alexander, 2015). The *in vitro* activity of ibrexafungerp is summarized in Table 1.

**Table 1:** *In vitro* activity of the main cell wall antagonists.

Species	Antifungal class			
	β-D-Glucan synthase inhibitors		Chitin synthase inhibitors	GPI anchor pathway inhibitors
	Echinocandins	Enfumafungin derivatives (Ibrexafungerp)	Nikkomycin Z	Fosmanogepix
*Candida* species	Strong	Strong	Poor but strong synergism with echinocandins	Strong
*Candida auris*	Strong	Strong	Not evaluated	Strong
*Aspergillus fumigatus*	Strong	Strong with synergism with azoles and amphotericin B	Poor	Strong
*Fusarium* species	Poor	Poor	Poor	Strong
*Lomentospora prolificans*	Poor	Moderate	Poor	Strong
*Coccidioides* species	Moderate^1^	Not evaluated	Moderate and with synergism with echinocandins	Strong
*Blastomyces dermatitidis*	Poor	Not evaluated	Moderate	Not evaluated
*Histoplasma capsulatum*	Poor	Not evaluated	Moderate	Not evaluated
*Cryptococcus* species	Poor	Poor	Poor but with strong synergism with azoles	Strong

*Strong in vitro activity was considered for the antifungals presenting minimal inhibitory concentrations, usually ≤0.5 mcg/μL for a certain genus or species; moderate in vitro activity was considered for the antifungals presenting minimal inhibitory concentrations usually between 0.5 and 4 mcg/μl for a certain genus or species; poor in vitro activity was considered for the antifungals presenting minimal inhibitory concentrations usually >4 mcg/μl for a certain genus or species*.

1 *Some studies described poor in vitro activity of echinocandins against Coccidioides spp. (Stevens, 2000; Cordeiro et al., 2006), while a recent study described strong in vitro activity of echinocandins against Coccidioides immitis (Thompson et al., 2017). The data presented in this table are based on the references: Aguilar-Zapata et al. (2015); Arendrup et al. (2018a); Shaw et al. (2018); Castanheira et al. (2012); Goldberg et al. (2000); Hage et al. (2011); Hector et al. (1990); Lamoth and Alexander (2015); Li and Rinaldi (1999); Nakai et al. (2003); Pfaller et al. (2019); Thompson et al. (2017); Viriyakosol et al. (2019); Zhao et al. (2018)*.

In time-to-kill experiments, ibrexafungerp showed mainly fungicidal activity against *Candida albicans* and *non-albicans* isolates (Scorneaux et al., 2017). For *in vivo* murine models of invasive candidiasis caused by *C. albicans*, *C. glabrata*, and *C. parapsilosis*, this drug showed similar concentration-dependent killing of the three Candida species (Lepak et al., 2015).

This antifungal is currently in clinical trials for the treatment of vulvovaginal candidiasis (Phase 3; https://clinicaltrials.gov/ct2/show/NCT03987620), for invasive aspergillosis in combination with voriconazole (Phase 2; https://clinicaltrials.gov/ct2/show/NCT03672292), invasive and mucosal candidiasis (Phase 3; https://clinicaltrials.gov/ct2/show/NCT03059992), and for invasive candidiasis due to *C. auris* (Phase 3; https://clinicaltrials.gov/ct2/show/NCT03363841).

### Chitin Synthase Inhibitors

Chitin is an essential component of the fungal cell wall and compounds that affect its synthesis have been investigated as antifungals, such as nikkomycins, polyoxins, and plagiochin (Chaudhary et al., 2013).

Nikkomycins are peptidyl nucleoside agents that competitively inhibit chitin synthase (*CHS*). Nikkomycin Z has some *in vitro* activity against *C. parapsilosis, Coccidioides immitis, and Blastomyces dermatitidis* (Hector et al., 1990), but its usefulness relies on the synergism with echinocandins for *C. albicans, A. fumigatus,* and *C. immitis* (Chiou et al., 2001; Cheung and Hui, 2017). One study using a murine model of invasive candidiasis showed that Nikkomycin Z plus echinocandins were effective for the treatment of infections by echinocandin-resistant *C. albicans* (Cheung and Hui, 2017).

### Glycosylphosphatidyl Inositol Anchor Pathway Inhibitors

Glycosylphosphatidyl inositol (GPI) is a component of the eukaryotes cell wall and is synthesized in the endoplasmic reticulum by a conserved pathway (Ikezawa, 2002). GPI glycolipids anchor different proteins to the cell wall and are essential for its integrity (Yadav and Khan, 2018).

Antifungals targeting GPI anchor synthesis pathway have been developed in the last 15 years (Tsukahara et al., 2003; Mutz and Roemer, 2016). One of the targets of the GPI anchor synthesis pathway is the protein Gwt1 (GPI-anchored wall protein transfer 1), an inositol acyltransferase that catalyzes inositol acylation (Tsukahara et al., 2003; Hata et al., 2011). Inhibition of Gwt1 compromises cell wall integrity, biofilm production, germ tube formation, and produces severe fungal growth defects (Yadav and Khan, 2018). In *C. albicans* and *Saccharomyces cerevisiae,* Gwt1 inhibition has been shown to jeopardize the maturation and stabilization of GPI-anchored mannoproteins (McLellan et al., 2012). The first compound used to inhibit the Gwt1 enzyme was the molecule 1-(4-butylbenzyl) isoquinoline (BIQ), described by Tsukahara et al. (2003).

From the BIQ molecule, a new compound with higher antifungal potency was created by the Tsukuba Research Laboratories of Eisai Co., Ltd. (Ibaraki, Japan), the APX001A or manogepix (formerly E1210) (Hata et al., 2011). Later, Amplix Pharmaceuticals Inc. (San Diego, CA, USA) developed the N-phosphonooxymethyl prodrug fosmanogepix (APX001, formerly E1211) with oral and IV formulations. The prodrug is metabolized by phosphatases and converted to manogepix (APX001A, formerly E1210) which inhibits the Gwt1 but not the human homolog Pig-W (Watanabe et al., 2012; Wiederhold et al., 2019). The oral formulation of fosmanogepix presented good bioavailability in murine experiments (Zhao et al., 2018).

The *in vitro* activity of manogepix has been investigated against yeasts and molds (Miyazaki et al., 2011; Castanheira et al., 2012). Low minimal inhibitory concentrations (MICs) of this new antifungal were found against *C. albicans, C. tropicalis, C. glabrata, C. parapsilosis, C. lusitaniae, C. kefyr,* (Miyazaki et al., 2011; Pfaller et al., 2019), and also against multidrug-resistant *C. auris* (Hager et al., 2018a), and echinocandin-resistant *C. glabrata* (Pfaller et al., 2019). However, *in vitro* results against *C. krusei* and *C. norvegensis* have been described as poor (Arendrup et al., 2018a). Potent *in vitro* activity of manogepix was also noticed against *Cryptococcus neoformans* and *Cryptococcus gattii* strains (Shaw et al., 2018; Pfaller et al., 2019). Regarding *in vitro* activity against molds, low MICs against *Aspergillus* species from the Section Fumigati, Flavi, Terrei, and Nigri (Miyazaki et al., 2011; Pfaller et al., 2019), *Purpureocillium lilacinum, Cladosporium* species, *Phialophora* species, *Rhinocladiella aquaspersa, Fonsecaea pedrosoi* (Miyazaki et al., 2011), *Scedosporium apiospermum*, and *Scedosporium aurantiacum* (Castanheira et al., 2012), and against the multidrug-resistant species *Fusarium solani* and *L. prolificans* (Castanheira et al., 2012). The *in vitro* activity of manogepix is summarized in Table 1.

The *in vivo* activity of manogepix/fosmanogepix has been also investigated in murine models of disseminated candidiasis, aspergillosis, fusariosis (Hata et al., 2011; Hager et al., 2018b), and *Coccidioides immitis* pneumonia (Viriyakosol et al., 2019). In a murine model of disseminated *C. albicans* infection, it showed similar efficacy to caspofungin, fluconazole, and liposomal amphotericin B (Hata et al., 2011). Another study compared the efficacy of manogepix/fosmanogepix and anidulafungin for the treatment of mouse with disseminated *C. auris* infection and found higher survival rates in the group treated with the Gwt1 inhibitor (Hager et al., 2018b). In a murine model of invasive *Aspergillus flavus* infection, mice treated with this new antifungal had similar survival rates when compared to the groups treated with either voriconazole or caspofungin (Hata et al., 2011). In the same study, mice infected by *F. solani* showed a higher survival rate when treated with fosmanogepix 20 mg/kg compared to the control group without antifungal therapy (Hata et al., 2011).

There is currently a Phase 2, single-arm, and open-label trial of fosmanogepix for the first-line treatment of candidemia^2^.

## Fungal Cell Wall Modifications and Antifungal Resistance

Modifications in fungal cell wall architecture appear after stresses produced by the host microenvironment and antifungal exposure (Ene et al., 2012; Perlin, 2015; Mesa-Arango et al., 2016).

*In vitro* studies have shown in conditions that mimic the host microenvironment at the infection site that yeast cells may develop wall modifications and antifungal resistance (Ene et al., 2012; Brown et al., 2014). *C. albicans* cells grown in serum (<0.1% glucose) show major changes in the cell wall architecture, with a decrease in the length of mannan chains, and in the chitin and β-glucan content (Ene et al., 2012). Moreover, growth-challenging conditions with alternative carbon sources, such as lactate, alter cell wall biosynthesis, leading to the production of a leaner but stiffer inner cell wall (Ene et al., 2012). These cell wall-remodeled *C. albicans* cells become resistant to amphotericin B (AMB) and caspofungin (Ene et al., 2012). Similar results were demonstrated for *C. glabrata* strains that grown under an alternative carbon microenvironment showed altered cell wall architecture with a lower content of chitin and β-glucan, and with an increased outer mannan layer (Chew et al., 2019). These *C. glabrata* cells were also resistant to AMB when grown in lactate or oleate (Chew et al., 2019).

An intermediary step to antifungal resistance is the development of tolerance (Perlin, 2015). Cells surviving drug exposure can respond to selection and evolve resistance (Perlin, 2015). Echinocandin exposure causes cell wall stress by inhibition of the β-D-glucan synthesis, which triggers adaptive cellular factors that stimulate chitin production (Walker et al., 2008, 2010). Protein kinase C (PKC), high osmolarity glycerol response (HOG), and Ca^+2^-calcineurin pathways have been implicated in the response to cell wall damage and chitin synthesis (Figure 1B; Lagorce et al., 2003; Bermejo et al., 2008; Walker et al., 2008; Fortwendel et al., 2009). The chaperone Hsp90 is another crucial component for echinocandins tolerance after cell wall stress (Singh et al., 2009; O’Meara et al., 2017). Calcineurin is a client protein for the Hsp90 chaperone and genetic compromise of the gene *HSP90* reduced the tolerance mechanism in *C. albicans* (Singh et al., 2009), *C. glabrata* (Singh-Babak et al., 2012), and *Aspergillus fumigatus* (Lamoth et al., 2014). Another expression of fungal adaptive mechanisms caused by antifungal stress is called the parodoxal effect, which is the recuperation of fungal growth after exposure to antifungals at increasing concentrations above a certain threshold (Aruanno et al., 2019). This phenomenon has been reported in *Candida* spp. and *Aspergillus* spp. after exposure to echinocandins, mainly caspofungin (Rueda et al., 2014; Marcos-Zambrano et al., 2017a; Aruanno et al., 2019). Similar to the tolerance mechanism, the paradoxical effect is related to intracellular signaling pathways that lead to cell wall remodeling with increase of the chitin and loss of β-D-glucan content (Aruanno et al., 2019). In *A. fumigatus*, caspofungin exposure may also lead to an increase in reactive oxygen species (ROS) production and to modifications of the lipid microenvironment surrounding the β-D-glucan synthase, leading to echinocandins resistance (Satish et al., 2019).

In *C. albicans*, other relevant components for echinocandin tolerance may be located at chromosome 5 (Ch5), since some tolerant mutants showed either monosomy of the Ch5, or combined monosomy of the left arm and trisomy of the right arm of Ch5 (Yang et al., 2017). Eventually, persistent echinocandin exposure leads to *FKS* mutations and organisms with marked and stable resistance emerge with a high chitin content in the cell wall (Walker et al., 2013; Perlin, 2015). *FKS* mutations in *Candida* species and echinocandin resistance have been extensively reviewed elsewhere (Walker et al., 2010; Perlin, 2015).

AMB resistance may be explained by multiple mechanisms, among them modifications in the cell wall architecture (Seo et al., 1999; Mesa-Arango et al., 2016). *Aspergillus flavus* isolates with AMB resistance have been related to invasive fungal infections with poor prognosis in neutropenic patients (Koss et al., 2002; Hadrich et al., 2012). Seo, Akiyoshi, and Ohnishi demonstrated that *in vitro* AMB-resistant mutant strains of *A. flavus* have similar sterol content in the cell membrane when compared to susceptible strains (Seo et al., 1999). Conversely, the cell wall from the resistant mutants contained more 1,3-β-D-glucan when compared to susceptible strains (Seo et al., 1999). The authors suggest that the higher content of glucans found in the resistant mutants helps to adsorb AMB, making it more difficult for the antifungal to reach the cell membrane (Seo et al., 1999). Comparisons between biofilm (AMB-resistant) and planktonic (AMB-susceptible) *C. albicans* cells revealed that the cell wall from the biofilm-grown isolates are thicker and have more β-1,3-glucans (Nett et al., 2007). In *C. tropicalis*, AMB resistance has been linked to several potential mechanisms, such as increase in catalase activity, changes in mitochondrial potential, low accumulation of reactive oxygen species, and deficiency in ergosterol at the cell membrane (Forastiero et al., 2013; Mesa-Arango et al., 2014). More recently, cell wall modifications have also been found in AMB-resistant *C. tropicalis* isolates (Mesa-Arango et al., 2016). The AMB-resistant isolates showed thicker cell walls with higher volume when compared to susceptible isolates (Mesa-Arango et al., 2016). Besides, these AMB-resistant organisms had a 2- to 3-fold increase of β-1,3-glucans in the cell wall (Mesa-Arango et al., 2016).

## Conclusions and Perspectives

Recent advances in the science of the fungal cell wall have opened the doors to new therapeutic modalities for fungal infections, and have helped to better understand the mechanisms of antifungal resistance. New antifungals targeting the cell wall show better safety and PK/PD profiles than the available toxic polyenes and azole derivative molecules. The new β-D-glucan synthase inhibitor ibrexafungerp has potent *in vitro* activity against multidrug-resistant pathogens such as echinocandin-resistant *C. glabrata*, *C. auris,* and *Aspergillus* species.

Glucan synthase inhibitors such as Nikkomycin Z have strong synergism with echinocandins and may be useful for the treatment of echinocandins-resistant *Candida* infections and refractory aspergillosis.

The GPI anchor pathway inhibitors APX001/APX001A have good pharmacokinetic profiles and strong *in vitro* activity against several fungal pathogenic species, including multiresistant *C. auris, F. solani,* and *L. prolificans*. This makes these drugs the most promising antifungals to be launched in the future.

The microenvironment at the infection site leads to modification in the fungal cell wall, which may lead to antifungal resistance. Cell wall stress induced by echinocandin exposure leads to the emergence of tolerant cells with high chitin content. The PKC, HOG, and Ca^+2^-calcineurin pathways, as well as the chaperone Hsp90, are crucial components for the phenomenon of antifungal tolerance and should be explored as future targets for antifungal therapy. A few AMB-resistant *A. flavus* and *C. tropicalis* showed higher content of glucans in the cell wall, but further studies analyzing the cell wall modifications and AMB resistance are necessary to increase the strength of this correlation.

## Author Contributions

SL, AC, and JA conceived of the manuscript. SL and JA conducted the literature review. SL, AC, and JA wrote the manuscript. AC revised the manuscript.

### Conflict of Interest

The authors declare that the research was conducted in the absence of any commercial or financial relationships that could be construed as a potential conflict of interest.

## Footnotes

^1^https://clinicaltrials.gov/ct2/show/NCT03667690

^2^https://clinicaltrials.gov/ct2/show/NCT03604705
